# Protein Profiling Reveals Novel Proteins in Pollen and Pistil of W22 (ga1; Ga1) in Maize

**DOI:** 10.3390/proteomes2020258

**Published:** 2014-05-05

**Authors:** Jin Yu, Swapan Kumar Roy, Abu Hena Mostafa Kamal, Kun Cho, Soo-Jeong Kwon, Seong-Woo Cho, Yoon-Sup So, James B. Holland, Sun Hee Woo

**Affiliations:** 1 Department of Crop Science, Chungbuk National University, Cheong-ju 361-763, Korea; E-Mails: edjlsa8603@naver.com (J.Y.); swapankhulna@gmail.com (S.K.R.); abuhena7@gmail.com (A.H.M.K.); kwonsj1220@naver.com (S.-J.K.); yoonsupso@cbnu.ac.kr (Y.-S.S.); 2 Division of Mass Spectrometry Research, Korea Basic Science Institute, Chungbuk 863-883, Korea; E-Mail: chokun@kbsi.re.kr; 3 Lab of Molecular Breeding, Arid land Research Center, Tottori University, Tottori 680-8550, Japan; E-Mail: whtjddn0435@chungbuk.ac.kr; 4 USDA-ARS Plant Science Research Unit, Department of Crop Science, Box 7620, North Carolina State University, Raleigh, NC 27695, USA; E-Mail: Jim.Holland@ars.usda.gov

**Keywords:** maize, pollen, pistil, gametophytic factors, proteomics

## Abstract

Gametophytic factors mediate pollen-pistil interactions in maize (*Zea mays* L.) and play active roles in limiting gene flow among maize populations and between maize and teosinte. This study was carried out to identify proteins and investigate the mechanism of gametophytic factors using protein analysis. W22 (ga1); which did not carry a gametophytic factor and W22 (Ga1), a near iso-genic line, were used for the proteome investigation. SDS-PAGE was executed to investigate proteins in the pollen and pistil of W22 (ga1) and W22 (Ga1). A total of 44 differentially expressed proteins were identified in the pollen and pistil on SDS-PAGE using LTQ-FTICR MS. Among the 44 proteins, a total of 24 proteins were identified in the pollen of W22 (ga1) and W22 (Ga1) whereas 20 differentially expressed proteins were identified from the pistil of W22 (ga1) and W22 (Ga1). However, in pollen, 2 proteins were identified only in the W22 (ga1) and 12 proteins only in the W22 (Ga1) whereas 10 proteins were confirmed from the both of W22 (ga1) and W22 (Ga1). In contrary, 10 proteins were appeared only in the pistil of W22 (ga1) and 7 proteins from W22 (Ga1) while 3 proteins confirmed in the both of W22 (ga1) and W22 (Ga1). Moreover, the identified proteins were generally involved in hydrolase activity, nucleic acid binding and nucleotide binding. These results help to reveal the mechanism of gametophytic factors and provide a valuable clue for the pollen and pistil research in maize.

## 1. Introduction

In angiosperms, pollen-pistil interactions are important for the subsequent successful reproduction and formation of seed [1]. In flowering plants, interaction between pollen and pistil ascertains reproductive compatibility [2]. Gametophytic factors are important, especially those known as pollen killer genes, gametocidal genes, gamete eliminators and gamete aborters. They have been introduced in several economically important plant species such as maize [3,4], tobacco [5], wheat [6], tomato [7], lima beans [8] and barley [9]. The first gametophytic factor (gametophytic factor 1) related to segregation distortion was reported in maize. However, the pollination with Ga1 pollen only or with ga1 pollen only, led to normal genotype ratios. Due to the fastening of pollen-tube growth in pollen with Ga1 than with ga1, a mixture of Ga1 and ga1 pollen resulted in an excess of the genotypes with the linked Su allele [3].

Maize (*Zea mays* L.) is a model species for investigating pollen-pistil interactions, and is one of the most essential cereal crops in the world [10]. However, several maize genotypes carry genes referred to as gametophytic factors that mediate pollen-pistil interactions and subsequently impair the success of fertilization [11]. Pollen-pistil interactions are essential for the seed and fruit formation, revealing that their mechanisms are of great importance, especially for understanding the completion of the plant life cycle and for accelerating agricultural production. Recent transcriptomic and proteomic studies have improved our knowledge regarding pollen/pistil gene and protein expression and eventually, the desirable genes are possibly involved in the pollen-pistil interactions [12].

The high-throughput proteomics approach is thought to be a powerful tool for the analysis of proteins related to gametophytic factors. The proteomes of pollen have been described previously [13,14,15,16,17,18], whereas the proteome analysis of pollen and pistil is relatively not well studied. The pollen and pistil protein of maize (*Zea mays* L.) were analyzed using SDS-PAGE combined with MS identification. However, two-dimensional gel electrophoresis (2-DE) combined with MS analysis have provided the most potential and reliable method for proteomic investigations. Previously, 2-DE techniques combined with MALDI-TOF (matrix assisted laser desorption ionization/time of flight) MS or ESI Q-TOF (electrospray ionization quadrupole-TOF) MS/MS have been executed to investigate the proteomes during pollen development within the anther as well as proteomes of mature and germinated pollen in various plant species [13,14,15,16,17,18]. These proteomic studies have significantly promoted our knowledge of the regulation of pollen and pollen tube development at the molecular level.

In addition, in F_1_ hybrid production, the use of gametophytic factors has played a crucial role because it prevents pollen from contamination. However, the mechanism of gametophytic factors is still unknown. For understanding the mechanism of gametophytic factors, proteomics has been employed to analyze proteins from pollen and pistil of maize. This study was carried out to identify proteins related to gametophytic factors using protein analysis.

## 2. Experimental

### 2.1. Plant Materials and Genetic Background

W22 (ga1, ga1) is a common inbred line developed by university of Wisconsin. It has a normal dent genotype; therefore, it does not carry a gametophytic factor. W22 (Ga1-s) line was created by Dr. Kermicle from university of Wisconsin by crossing with a popcorn (white cloud variety) which carries Ga1-s to W22, followed by five successive backcrossing to W22, while selecting for Ga1-s.

Seeds of W22 (ga1) and W22 (Ga1) were sown in a greenhouse in the seedling tray. After 3~4 days, it was transplanted to 6 pots and the paper bag was covered to prohibit fertilization between the pollen and pistil of same maize line during anthesis period. Within a week, one gram of maize pollen and pistil were incubated in 50 mL of 0.1 M NH_4_HCO_3_ buffer (pH 8.0) for 30 min. Then, the soluble fraction was isolated by centrifugation at 17,000× *g* for 30 min and dialyzed against double-distilled water overnight. The extract was lyophilized and stored at 4 °C for further use. All experiments are replicated 3 times.

### 2.2. Protein Extraction and Electrophoresis

A portion (0.5 g) of pollen and pistil was ground in liquid nitrogen. Using a modified method, the proteins were extracted from the pollen and pistil according to previously described methods [19]. The seeds were then suspended in Solution I [(10% trichloroacetic acid (TCA) in acetone containing and 0.07% 2-mercaptoethanol (2-ME)] and then sonicate for 5–10 min. Solution II [0.07% 2-mercaptoethanol (2-ME) in acetone containing] was added in the pellets and the vortex, and then centrifuged at 20,000× *g* at 4 °C for 5 min. This step was repeated and the pellets were dried by vacuum centrifugation for 10 min. The dried powder was diluted with lysis buffer (7 M urea, 2 M thiourea, 5% CHAPS, and 2 mM tributylphosphine), incubate at 37 °C for 2 h and then centrifuged at 20,000× *g* at 4 °C for 20 min. The supernatants were collected to 1.5 mL tube. The protein concentrations were determined by RC/DC assay and then it was stored at −80 °C for further utilization.

Proteins were extracted from the pollen and pistil according to TCA/acetone precipitation method prior to SDS-PAGE. Proteins were separated on 16 × 16 cm SDS-PAGE gels (gradient 14%–16% acrylamide) as described previously [20]. The electrophoresis conditions were set and run at 50 m. A for 2 h until the sample buffer dye reached the lower part of the gel. The experiment is biologically triplicate. The gels were stained with coomassie brilliant blue R-250 and scanned using a scanner (HP Scanjet G4010, Palo Alto, CA, USA).

### 2.3. In-gel Digestion

CBB-stained gel slices were washed several times with 30% methanol until the colors were completely removed. Then the gel slices were destained with 10 mM NH_4_HCO_3_ in 50% ACN (Acetonitrile), squeezed for 10 min with 100% ACN (Acetonitrile) and dried by vacuum centrifugation. After destaining steps, the gel slices were reduced with 10 mM DTT in 100 mM NH_4_HCO_3_ at 56 °C for 1 h and then alkylated with 55 mM Iodoacetamide (IAA) in 100 mM NH_4_HCO_3_ in the dark for 40 min. Then the gel slices were digested with 50 μL trypsin (10 ng/µL) (Promega Corporation, Madison, WI, USA) and incubated at 37 °C for 16 h. After digestion steps, the peptides were extracted with 50 mM ammonium bi-carbonate and repeated these steps several times with a solution containing 0.1% formic acid in 50% ACN (acetonitrile) until 200~250 μL. The solution containing eluted peptides was concentrated up to drying by vacuum centrifugation and the resultant extracts were confirmed by LTQ-FTICR mass spectrometry. The dried samples were stored at 4 °C prior to mass spectrometry analysis.

### 2.4. MS/MS Analysis and Bioinformatics

All MS experiments for peptide identification were performed on a Nano-LC/MS system consisting of a Surveyor HPLC system and a 7-tesla Finningan LTQ-FTICR mass spectrometer (Therm Electron, Bremen, Germany) equipped with a nano-ESI source. Ten microliters of each sample were loaded by a Surveyor auto sampler (Surveyor) onto a C18 trap column for desalting and concentration at a flow rate of 20 μL/min. The mass spectrometer was operated in the data-dependent mode to automatically switch between MS and MS acquisition. General mass spectrometric conditions included spray voltage, 2.2 kV; no sheath and auxiliary gas flow; ion transfer tube temperature, 220 °C; collision gas pressure, 1.3 millitorrs; normalized collision energy using wide band activation mode; and 35% of MS. Ion selection threshold was 500 counts for MS/MS. An activation *q* = 0.25 and an activation time of 30 ms were applied in MS/MS acquisitions. Acquired MS spectra were searched using an in-house licensed MASCOT search engine (Mascot version 2.2.04; Matrix Science, London, UK). To identify the peptides, MASCOT (version 2.3.01, Matrix Science, London, UK), operated on a local server, was used to search the maize (*Zea mays*) database. MASCOT was used to the monoisotopic mass selected, a peptide mass tolerance of 10 ppm, and a fragment ion mass tolerance of 0.8 Da. Trypsin was selected as enzyme, with one potential missed cleavage. ESI-FTICR was selected as instrument type, and carbamidomethyl cysteine and oxidized methionine were chosen as variable modifications. All proteins identified by high-scoring peptides were considered true matches, and at least two peptide matches. The high-scoring peptides corresponded with the peptides that were above the threshold in our MASCOT search (expected *p* < 0.05).

## 3. Results and Discussion

### 3.1. Protein Expression on SDS-PAGE

**Figure 1:**
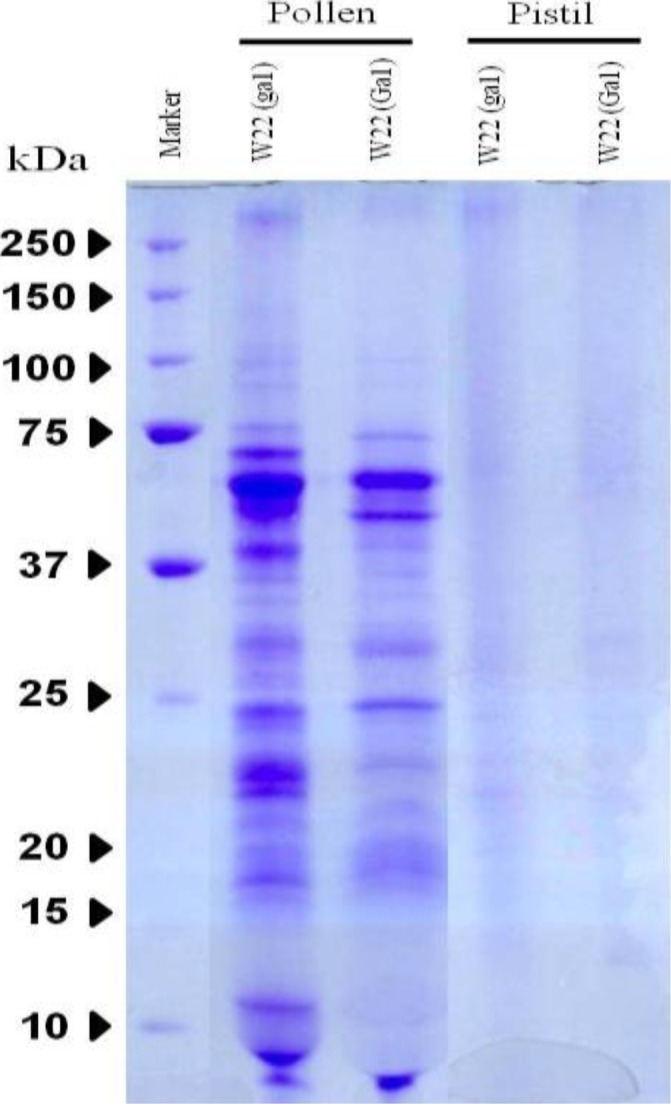
SDS-PAGE pattern in the pollen and pistil of W22 (ga1; Ga1) in maize. Samples were analyzed triplicate as described in the method section and gels were stained using Coomassie Brilliant Blue (CBB) staining. Standard molecular weight (kDa) is on the left.

SDS-PAGE was performed in order to profile proteins from the pollen and pistil. Pollen and pistil were collected from wild type W22 (ga1) and near-isogenic lines W22 (Ga1), respectively. Pollen lanes were well separated whereas pistil lanes were not clearly visual (Figure 1, Supplementary Figure 1). However, high performance LTQ-FTICR MS was used to excise the gel into 10 pieces.

### 3.2. Specific Protein Analysis of Identifying Proteins from Pollen

Twenty four differential expressed proteins were identified from pollen on SDS-PAGE using LTQ-FTICR MS. Two proteins namely chaperonin CPN60-2 and albumin b-32 were only identified from the pollen of W22 (ga1), whereas 12 proteins were only appeared in the pollen of W22 (Ga1) such as adagio protein 3, ATP synthase subunit alpha, ATP synthase subunit beta, histone H2B.4, 1-Cys peroxiredoxin PER1, glucose-6-phosphate isomerase, ADP, ATP carrier protein 2, cysteine synthase, ferredoxin-dependent glutamate synthase, expansin-B9, expansin-B1, peptidyl-prolyl *cis*-trnas isomerase (Table 1). However, 10 proteins were commonly shared from both of the W22 (ga) and W22 (Ga1) like elongation factor 1 alpha, exopolygalacturonase (3 subunits), expasin B-10, profilin-3, endochitinase A, endochinase B, expansin B-11 and ribosome-inactivating protein 3.

### 3.3. Specific Protein Analysis of Identified Proteins from Pistil

Out of 20 proteins, 10 proteins (catalase isozyme 1, catalase isozyme 3, acetolactate synthase 2, ADP-ATP carrier protein 1, asparagine synthetase, histone H2B.1, histone H2A, histone-lysine *N*-methyltransferase EZ1, 14-3-3-like protein GF14 12, polycomb group protein FIE2) were identified in the pistil of W22 (ga1), where as 7 proteins (14-3-3 like protein GF14-6, 3-hydroxy-3-methylglutaryl-coenzyme A reductase, Glyceraldehyde-3-phosphate dehydrogenase A, Glyceraldehyde-3-phosphate dehydrogenase, casein kinase II subunit alpha, late embryogenesis abundant protein EMB564, Cell number regulator 2) were identified in the pistil of W22 (Ga1) (Table 1). However, 3 proteins like 2,3-bisphosphoglycerate-independent phosphoglycerate mutase, alpha-amylase/trypsin inhibitor, histone-lysine *N*-methyltransferase EZ1 were identified in both of the pistil of W22 (ga1) and W22 (Ga1) (Table 1).

**Table 1:** Features of the identified proteins in the pollen and pistil of W22 (ga1; Ga1) using Linear Quadruple Trap-Fourier-Transform Ion Cyclotron Resonance mass spectrometer (LTQ-FTICR-MS).

AN ^1^	Protein Description	W22 (ga1)		W22 (Ga1)		PS ^2^	MW^.3^	PM ^4^	p*I*^.5^	PC^.6^	MS-MS Ion Score
		Pollen	Pistil	Pollen	Pistil						
**Hydrolase activity**											
P26216	Exopolygalacturonase	√	×	√	×	79	43,416	6.95	4	11.7	64.93
P35339	Exopolygalacturonase	√	×	√	×	63	43,269	8.44	5	17.8	67.85
P35338	Exopolygalacturonase	√	×	√	×	79	43,387	6.59	5	15.1	66.03
Q41803	Elongation factor 1-alpha	√	×	√	×	96	49,202	9.19	7	23	46.8
P29022	Endochitinase A	√	×	√	×	103	29,106	8.3	3	22.5	69.56
P29023	Endochitinase B (Fragment)	√	×	√	×	61	28,147	8.94	2	14.9	59.82
P10593	Albumin b-32	√	×	×	×	36	32,408	5.38	3	20.8	46.86
P25891	Ribosome-inactivating protein 3	√	×	√	×	30	33,236	5.83	3	15.3	36.78
**Nucleotide binding**											
Q43298	Chaperonin CPN60-2, mitochondrial	√	×	×	×	31	60,897	5.67	2	6.2	9.26
P49094	Asparagine synthetase	×	√	×	×	21	66,535	5.83	2	6.1	21.43
P05494	ATP synthase subunit alpha, mitochondrial	×	×	√	×	102	55,146	8	5.85	19.3	34.9
P49106	14-3-3-like protein GF14-6	×	×	×	√	157	29,644	3	4.76	17.6	48.74
O24594	3-hydroxy-3-methylglutaryl-coenzyme A reductase	×	×	×	√	28	60,892	2	6.77	6	3.07
**Nucleic acid binding**											
P30755	Histone H2B.1	×	√	×	×	32	16,410	10	5	39.1	16.68
P40280	Histone H2A	×	√	√	×	65	16,417	10.59	2	28.3	70.53
Q8S4P5	Histone-lysine N-methyltransferase EZ2	×	√	×	√	24	99,916	8.47	7	11	9.82
Q8S4P6	Histone-lysine N-methyltransferase EZ1	×	√	×	×	14	103,703	8.85	3	4.7	2.64
P49120	Histone H2B.4	×	×	√	×	65	15,173	4	10.02	31.4	4.97
**Catalytic activity**											
P80608	Cysteine synthase	×	×	√	×	31	34,185	2	5.91	10.5	5.12
P23225	Ferredoxin-dependent glutamate synthase, chloroplastic	×	×	√	×	18	175,063	5	6.21	5.8	8.66
Q41769	Acetolactate synthase 2	×	√	×	×	31	68,982	6.48	3	9.1	29.41
P30792	2,3-bisphosphoglycerate-independent phosphoglycerate mutase	×	√	×	√	59	60,582	5.29	3	9.7	67.15
**Antioxidant activity**											
P18122	Catalase isozyme 1	×	√	×	×	27	56,841	7.4	2	9.8	26.82
P18123	Catalase isozyme 3	×	√	×	×	27	56,760	6.47	2	4.8	5.02
A2SZW8	1-Cys peroxiredoxin PER1	×	×	√	×	28	24,890	2	6.31	10.5	38.51
**Hydrolase activity**											
P04709	ADP, ATP carrier protein 1	×	√	×	×	41	42,365	9.85	4	11.1	22.01
857	ADP, ATP carrier protein 2	×	×	√	×	56	42,306	4	9.85	12.4	4.23
**Protein binding**											
P35083	Profilin-3	√	×	×	×	60	14,228	4.91	2	29	60.41
Q01526	14-3-3-like protein GF14-12	×	√	×	×	149	29,618	4.75	3	18	8.99
**Oxidoreductase activity**											
P09315	Glyceraldehyde-3-phosphate dehydrogenase A, chloroplastic	×	×	×	√	31	42,840	2	7	8.7	30.94
P08735	Glyceraldehyde-3-phosphate dehydrogenase, cytosolic 1	×	×	×	√	44	36,500	3	6.46	13.9	5.2
**Transporter activity**											
P19023	ATP synthase subunit beta, mitochondrial	×	×	√	×	128	59,067	8	6.01	25.9	25.94
P28523	Casein kinase II subunit alpha	×	×	×	√	16	39,205	3	8.41	14.8	16.2
**Isomerase activity**											
P21569	Peptidyl-prolyl cis-trans isomerase	×	×	√	×	127	18,337	2	8.91	18	36.38
P49105	Glucose-6-phosphate isomerase	×	√	√	×	36	62,198	2	6.96	4.1	1.62
**Enzyme regulator activity**											
P13867	Alpha-amylase/trypsin inhibitor	×	√	×	√	64	22,060	8.16	2	15	62.82
**Signal transducer activity**											
Q9C9W9	Adagio protein 3	×	×	√	×	23	69,019	3	6.06	2.3	9.6
**Unknown**											
P0C1Y5	Expansin-B11	√	×	√	×	174	28,943	8.44	4	13.8	39.64
Q8VZY6	Polycomb group protein FIE2	×	√	×	×	27	42,475	5.89	2	15.3	9.65
Q07154	Expansin-B9	×	×	√	×	200	29,062	5	9.01	21.6	39.5
P58738	Expansin-B1	×	×	√	×	31	29,066	4	8.99	18.6	39.5
P46517	Late embryogenesis abundant protein EMB564	×	×	×	√	55	9678	2	6.6	27.5	55.46
B6TYV8	Cell number regulator 2	×	×	×	√	18	19,222	2	7.37	17.7	18.17

^1^ AN: Accession Number, ^2^ PS: Protein Score, ^3^ MW: Molecular Weight, ^4^ PM: Protein Matches, ^5^ p*I*: Iso-electric Point, ^6^ PC: Protein Coverage.

### 3.4. Cross-Correlation and Functional Distribution of Identified Proteins from Pollen and Pistil

**Figure 2:**
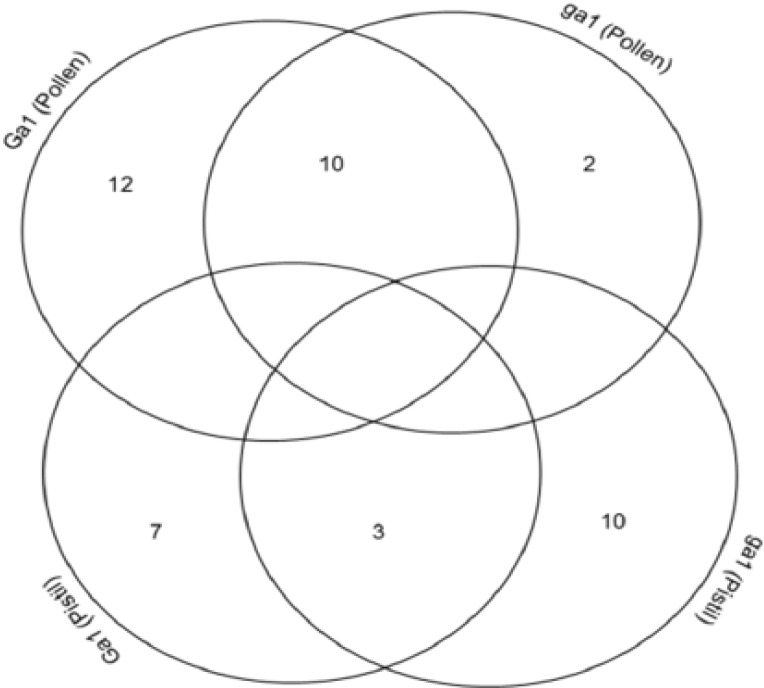
Cross-correlation of the identified protein between pollen and pistil of W22 (ga1; Ga1) in maize.

The cross-correlation was clarified of total identified proteins between pollen and pistil. Two proteins were identified from the pollen of W22 (ga1) whereas 12 proteins from the pollen of W22 (Ga1). However, 10 proteins shared from both of the pollen of W22 (ga1) and W22 (Ga1) (Figure 2). Furthermore, 10 proteins were identified from the pistil of W22 (ga1) whereas 7 proteins confirmed from the pistil of W22 (Ga1). However, 3 proteins shared from both of the pistil of W22 (ga1) and W22 (Ga1) (Figure 2).

**Figure 3:**
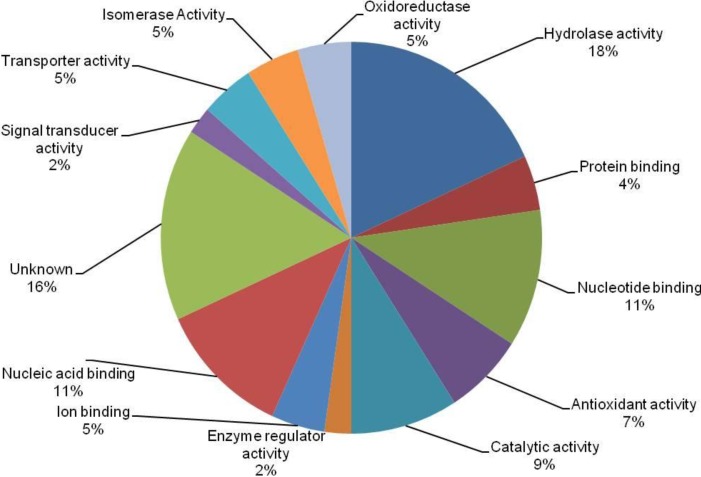
Functional classification of the total identified proteins in the pollen and pistil of W22 (ga1; Ga1) in maize.

A total of 44 differentially expressed proteins were classified into 13 possible functional categories by using Protein Information Resources (PIR) shown in Figure 3. Out of 44 unique proteins, most of them involved in hydrolase activity (18%), nucleotide binding (11%), nucleic acid binding (11%), catalytic activity (9%), antioxidant activity (7%), isomerase activity (5%), oxidoreductase activity (5%), transporter activity (5%), ion binding (5%), protein binding (4%), enzyme regulator activity (2%), signal transducer activity (2%) and unknown (16%) (Figure 3). 

### 3.5. The Implication of Differentially Expressed Proteins from Pollen and Pistil of Maize

A total of 44 proteins were identified from the pollen and pistil of W22 (ga1) and W22 (Ga1) of which 24 proteins were confirmed from the pollen of W22 (ga1) and W22 (Ga1) and 20 proteins from the pistil of W22 (ga1) and W22 (Ga1). However, two proteins were identified from the pollen of W22 (ga1) whereas albumin b-32 (32.4 kDa, p*I* 5.38) regarded as the protein of maize endosperm that is a monomeric albumin with an apparent molecular weight of about 32 kDa with a p*I* of 5.38. Di Fonzo *et al.*, 1988 [21] found that the two variants expose similar amino acid composition but minor differences are appeared by their tryptic peptide maps. They also noticed that the protein is localized in the soluble part of the cytoplasm and does not bind to any particular structure.

In addition, 12 proteins were identified only from the pollen of W22 (Ga1). However, ATP synthase subunit alpha (55.1 kDa, p*I* 5.85) and ATP synthase subunit beta (59.0 kDa, p*I* 6.01) were confirmed in our investigation. ATP synthesis is membrane-bound enzyme complexes/ion transporters that accelerate ATP synthesis and/or hydrolysis with the transport of protons across a membrane. It can harness the energy from a proton gradient, using the flux of ions across the membrane via the ATPase proton channel to drive the synthesis of ATP. The alpha/A and beta/B subunits can each be divided into three regions, or domains, centered on the ATP-binding protein, and based on structure and function. The central domain contains the nucleotide-binding residues that make direct contact with the ADP/ATP molecule [22]. 1-Cys peroxiredoxin PER1 (24.8 kDa, p*I* 6.31) was considered as the antioxidant protein which seems to contribute to the inhibition of germination during stress. It was prevailed that overexpression of rice 1-cys-peroxiredoxin in transgenic tobacco accelerated oxidative stress tolerance, but dormancy was not affected [23]. Glucose-6-phosphate isomerase (62.1 kDa, p*I* 6.96) was identified in the pollen of W22 (Ga1) that catalyzes the conversion of glucose-6-phosphate into fructose 6-phosphate in the second step of glycolysis. This protein has various functions inside and outside the cell. This protein is also involved in the glycolysis and gluconeogenesis within the cytoplasm, while outside the cell it acts as a neurotrophic factor for spinal and sensory neurons. In *Ananas comosus*, it was prevailed that the mitochondria may produce this protein to allow cytoplasmic conversion of glucose-6 phosphate into fructose-6 phosphate in the second step of glycolysis [24]. ADP, ATP carrier protein 2 (42.3 kDa, p*I* 9.85) was found in the pollen of W22 (Ga1) that catalyzes the exchange of ADP and ATP over the mitochondrial inner membrane. An ADP/ATP carrier protein was found in *K. pinnata* mitochondria. The protein may be employed in the mitochondrial energy synthesis in which ATP synthase provides ATP via oxidative phosphorylation, and may work in reverse as a proton-pumping ATPase. It was revealed at *K. pinnata* that ADP and ATP could sustain via ADP/ATP carrier proteins between mitochondrial membranes and other organelles [24]. Cysteine synthase (CS) was identified in the pollen of W22 (Ga1) with molecular weight 34.1 kDa and p*I* 5.91. CS catalyzes the biosynthesis of cysteine in plants [25]; cysteine acts as a precursor for the synthesis of various sulfur containing metabolites [26], whereas glutathione represents the most important one which employed as a universal antioxidant and detoxifier for coping with various stresses [27]. Ferredoxin-dependent glutamate synthase (175 kDa, p*I* 6.21) was identified in the pollen of W22 (Ga1) that is involved in metabolic function especially in nitrogen assimilation. In Arabidopsis, the increase of ferredoxin dependent glutamate synthase is probably a consequence of limited electron transport and may affect feedback regulation to compete for electrons required for nitrogen assimilation [28].

In pistil, 10 proteins were identified in the W22 (ga1). However, catalase isozyme 1 (24.8 kDa, p*I* 6.31) was detected in the W22 (ga1) that occurs in almost all aerobically respiring organisms and serves to protect cells from the toxic effects of hydrogen peroxide. In the early stage of drought stress, catalase activities were found to increase or be stable, and then decrease with further increase in magnitude of water stress [29]. Furthermore, catalase isozyme 1 was only increased at 10 DPA under drought stress in Kauz which indicated that catalase might be activated to diminish toxic compounds during the early stage while the plant acclimatize the drought stress [30]. However, the catalase isozyme 3 (56.7 kDa, p*I* 6.47) was identified in the pistil of W22 (ga1) and it occurs also in almost all aerobically respiring organisms and serves to protect cells from the toxic effects of hydrogen peroxide. Its levels are highest in the light period and are lowest in the dark period. Therefore, it may be important for scavenging hydrogen peroxide at night, rather than during the day. Acetolactate synthase 2 (68.9 kDa, p*I* 6.48) was confirmed in the pistil of W22 (ga1). The acetolactate synthase (ALS) enzyme is a protein observed in plants and micro-organisms. ALS catalyzes the first step in the synthesis of the branched-chain amino acids (valine, leucine, and isoleucine) [31]. This protein is well known enzyme that is involved in catalytic activity, especially a part of the biosynthesis of various amino acids. However, in plants, it is located in the chloroplasts to assist the metabolic processes. It has been found in several experiments that mutated strands of *Escherichia coli* K-12 without the enzyme were not able to grow in the presence of only acetate as the only carbon sources [32]. Asparagine synthetase (66.5 kDa, p*I* 5.83) is an enzyme that generates asparagine from aspartate and arises only in the pistil of W22 (ga1). This reaction is similar to that accelerated by glutamine synthetase. It is also possible that asparagine synthetase poses its effects by fulfilling an as yet unknown function in the cell that is independent of its catalytic activity [33]. 14-3-3 like protein GF14-6 (29.6 kDa, p*I* 4.76) was confirmed in the pistil of W22 (ga1) that is associated with a DNA binding complex to bind to the G box, a well-characterized *cis*-acting DNA regulatory element found in plant genes. The functional properties of 14-3-3s are to bind and activate tyrosine and tryptophane hydroxylase in bovine brain in the presence of Ca^2+^/calmodulin-dependent protein kinase type II [34].

However, seven proteins were detected only in the pistil of W22 (Ga1). Glyceraldehyde 3-phosphate dehydrogenase (36.4 kDa, p*I* 7.01) were identified from the pistil of W22 (ga1) that catalyzes the conversion of glyceraldehyde 3-phosphate to D-glycerate 1, 3-bisphosphate. In soybean, glyceraldehyde 3-phosphate dehydrogenase (GAPDH) was identified as down-regulated at both the mRNA and protein levels in response to NaCl treatment, suggesting that it plays a role in salt stress and can be used as a target gene in soybean seedlings [35]. The main role of this gene is the tolerance and its relationship to improving salt tolerance in plants [36]. It is revealed that the ATP production will be reduced by the down-regulation of glyceraldehydes-3-phosphate dehydrogenase and eventually there will be a decrease in plant growth under salt stress. Late embryogenesis abundant protein EMB564 (9.6 kDa, p*I* 6.6) constitutes a set of proteins that participate in plant stress responses. During exposure to abiotic challenges, late embryogenesis abundant (LEA) proteins accumulate naturally in desiccation-tolerant structures, such as seed or pollen grains, and in plant vegetative tissues. However, Emb564 acts for displaying a complex combination of different PTMs, including phosphorylation, acetylation, methylation and deamination in the native protein, which may be relevant for its seed-specific role [37].

Furthermore, 10 proteins were detected from the pollen which shared both W22 (ga1) and W22 (Ga1) and three proteins were shared from both W22 (ga1) and W22 (Ga1) of pistil. Profilin-3 (14.2 kDa, p*I* 4.91) was confirmed in the pollen of both W22 (ga1) and W22 (Ga1). Profilins generate a large and diverse protein family. Multiple isoforms of profilins are available in many species, being encoded by separate genes, or in some cases translated from mRNA splice variants. In the case of animals and higher plants, isoforms may be exposed in a tissue-specific manner. Moreover, profilins are identified at different subcellular locations [38]; in particular, enrichment of the dynamic plasma membranes was ascertained for various cells types. Also, profilins were investigated in association with internal membranes that implicated in vesicular transport [39]. It revealed that the overall functional properties of different profilins are similar and eventually, one isoform can be interchanged with another one from quite a distant source [40]. Endochitinase A (29.3 kDa, p*I* 8.3) was identified from both of the pollen of W22 (ga1) and W22 (Ga1) that defends against chitin containing fungal pathogens. In *Arabidopsis thaliana*, a basic endochitinase (At3g12500) was confirmed that was involved in the ethylene/jasmonic acid-mediated signaling pathway during systemic acquired resistance [41].

## 4. Conclusions

The protein analysis of pollen and pistil in maize was accomplished to profile proteins related to gametophytic factors. Using SDS-PAGE, a total of 24 proteins from pollen and 20 proteins from pistil were identified following LTQ-FTICR MS. However, 2 proteins were only found in the pollen of W22 (ga1) whereas 10 proteins were revealed in the pollen of W22 (Ga1). In the case of the pistil, 10 proteins appeared in W22 (ga1) and 7 proteins were distinctly observed in W22 (Ga1). The proteins were mostly involved in the hydrolase activity, nucleic acid binding and nucleotide binding. More extensive studies are needed to fully understand the mechanism of gametophytic factors underlying the pollen and pistil of the lines.

## Supplementary Materials

Supplementary File 1
