# Supplementary material for: Protein Profiling Reveals Novel Proteins in Pollen and Pistil of W22 (ga1; Ga1) in Maize
Source: Proteomes. 2014 May 5;2(2):258–71. doi: 10.3390/proteomes2020258 (PMC5302736; doi:10.3390/proteomes2020258)

Supplementary Material for

Protein Profiling Reveals Novel Proteins in Pollen and Pistil of W22 (ga1; Ga1) in Maize

Supplementary Figure S1. The triplicate SDS-PAGE pattern in the pollen and pistil of W22 (ga1; Ga1) in maize. The gels were stained using CBB staining. Standard molecular weight (kDa) is on the left.

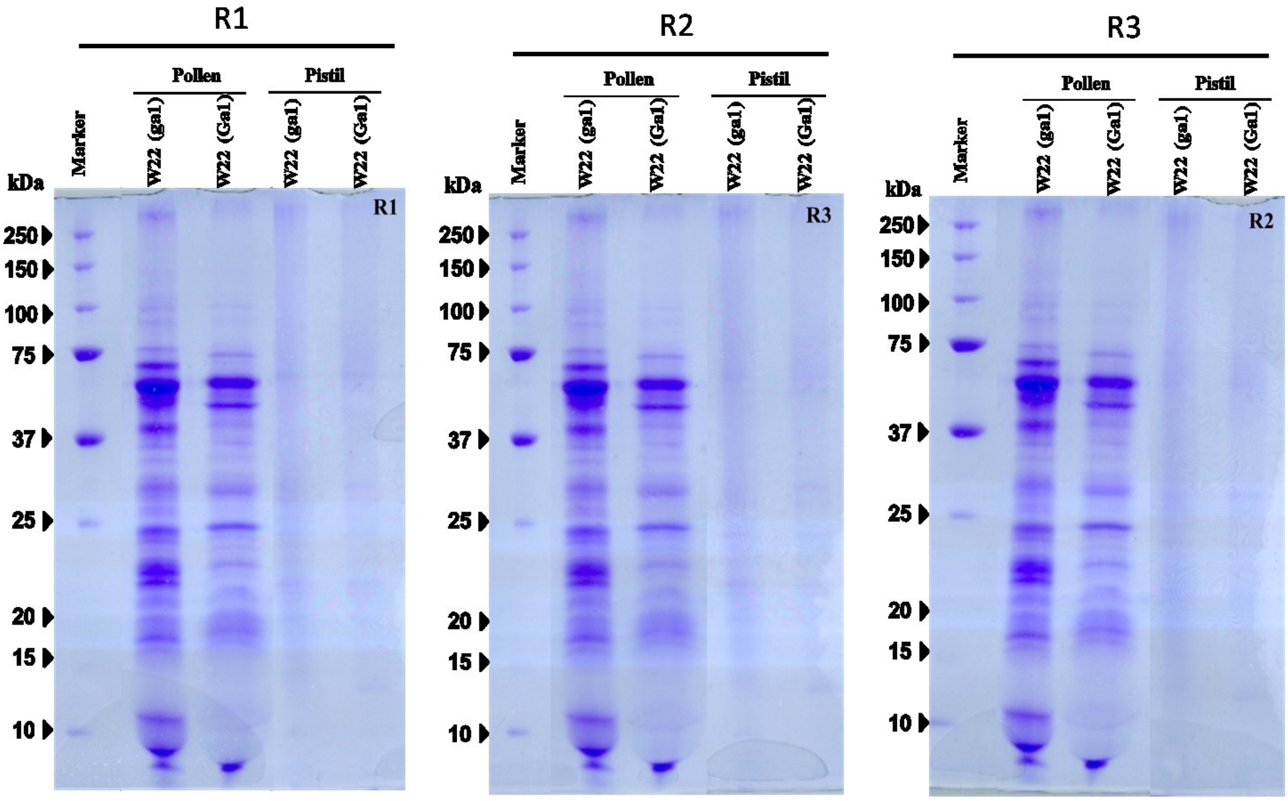

Supplement: Supplementary File 1 [file proteomes-02-00258-s001.pdf]
